# Intelligence

**Published:** 1832-08

**Authors:** 


### INTELLIGENCE.

Anatomy Bill. This Bill was read a third time, and passed, on Thursday the 19th, in the House of Lords. The numbers were twenty-nine for, and nine against it.

College of Surgeons. Mr. Vincent has been appointed president, and Mr. Guthrie and Mr. White vice-presidents for the ensuing year.

### BRITISH ASSOCIATION FOR THE ADVANCEMENT OF SCIENCE.

This Association, instituted last year at York, held its second annual meeting at Oxford during the week commencing the 18th June; and a week more full of interest and pleasure we scarcely remember ever to have passed. The manner in which the Association was received by the University was most gratifying to the one and honourable to the other. Every accommodation and convenience that Oxford could afford was most freely and nobly given; given with that hearty welcome which increased the value tenfold.

The arrangements made by the committee for the despatch of business were very judicious, and to their labours, which must have been extreme; the members were greatly indebted for the facility with which all that was going on became known, and admission obtained to all or any of the meetings. Some slight improvements may probably be suggested for future sessions, but, on the whole, the present arrangements were highly satisfactory.

To trace even in outline the transactions of the several days, if not requiring ubiquity, would at least demand the eyes and hands of Argus and Briareus: we can therefore but faintly sketch some few of the more important.

On Monday the committee met at eleven A.M., to arrange preliminary business; at twelve a general meeting took place, for the election of new members, and we hear that between four and five hundred joined the Association while at Oxford. The continued arrivals during the day, coaches and carriages of all descriptions being loaded to excess, caused much general bustle, but prevented any scientific matters being commenced until the evening, when the members met at the Clarendon buildings, which have lately been fitted up for the use of the University, and which were for the week surrendered to the Association.

On Tuesday morning the Society met at the Clarendon in sections, to receive and to read reports and communications. The meetings here were held in sections and rooms appropriated to each, so that the members could attend any one, or go occasionally to all, as best suited their respective tastes. The four chief sections were

A. Mathematical and physico-mathematical science, (astronomy, mechanics, hydrostatics, hydraulics, light, heat, sound, and meteorology.)

B. Chemistry, electricity, galvanism, magnetism, mineralogy, and chemical arts and manufactures.

C. Geology and geography.

D. Natural history, (botany, zoology, physiology, anatomy, medicine, &c.)

At these sectional meetings, which were continued daily (with the exception of Thursday,) from ten to half-past twelve, many papers and reports of extreme interest were read, and upon them some very animated discussions ensued, the importance of which may be well admitted when we mention that the following, with many other philosophers, were very constant in their attendance, and entered fully into the spirit of the respective subjects.

1. *Mathematics and general physics.* Professors Airy, Babbage, Powel, Hamilton; Sirs David Brewster, T. Brisbane; Drs. Davies, Gilbert, Pearson; Messrs. Whewell, Willis, Walker, &c.: Dr. D. Gilbert being chairman, and the Rev. H. Coddrington secretary of this section.

2. *Chemistry and mineralogy.* Professors Daniell, Cumming, Turner, Miller, Ritchie, Danberry; Drs. Prout, Gregory, Dalton, Faraday; Messrs. Johnston, Harris, Scoresby, Guillemard, &c.: Dr. Dalton being the chairman, and Mr. Johnston the secretary of this section.

3. *Geology and Geography.* The Marquess of Northampton, Viscount Cole, Professors Buckland and Sedgwick; Drs. Fillon and Turner; Messrs. Murchison, William Smith, Gideon Mantell, and others: Mr. Murchison being chairman, and Mr. Taylor secretary of this section.

4. *Natural history.* Professors Williams, Henlow, and Burnett; Drs. Kidd, Brown, Danberry, and Prichard; Messrs. Duncan, Vigors, Sabine, Clift, Broughton, Burchell, &c.: Mr. P. Duncan being chairman, and Professor Henslow secretary of this section.

At one o'clock on Tuesday, a general meeting was held in the Sheldonian Theatre, Lord Milton taking the chair, as president of last session, for the purpose of introducing Dr. Buckland as president elect, to whom it was immediately resigned.

Dr. B., in an eloquent address, descanted upon the advantages likely to result from such an association, and concluded by calling on Professor Airy for his report on the progress of astronomy; and a most admirable report it was. We trust it will be printed entire, as, from its length, several sections were obliged to be omitted in the perusal.

The second report was written by Mr. Lubbock, but, on account of his absence, committed to Mr. Whewell, who, having been pursuing similar researches in the natural history of the tides, added many of his own observations to those of Mr. Lubbock; and their philosophy of the progress of the tides and doctrine of co-tidal lines, as thus explained, gave great satisfaction, much of the matter being new, and the whole very important.

At five o'clock the visiters were invited to dinner by the resident members in the hall of New College, where covers were laid for nearly three hundred; Dr. Buckland in the chair, supported by the Marquess of Northampton and Lord Milton. The entertainment went off with much spirit; almost all the philosophic societies in the kingdom being toasted, and the president (we believe) making, during the day, seven and thirty speeches. About ten o'clock the members adjourned to the Clarendon, and a demonstration was given by Mr. Henning of the power of his newly-invented safety oxy-hydrogen blowpipe.

On Wednesday morning, by invitation of the Vice-chancellor, the members of the Association breakfasted in the hall and gardens of Exeter College. We should think about four hundred persons partook of the reverend Doctor's hospitality.

The sectional meetings, as the Clarendon, and the general in the theatre, were this day counterparts of Tuesday's proceedings. The most interesting occurrence was certainly the presentation of Dr. Wollaston's medal, by the president of the Geological Society, to Mr. William Smith, the father of English geology. The reports read were those by Professor Cumming on Thermo-electricity, and Mr. Forbes on Meteorology; a lecture was also given by Mr. Willis on the Philosophy of Sound. Dr. Kidd and several other persons gave dinners on this day to numerous parties; and in the evening two lectures were delivered in the music-room, one by Professor Ritchie on Faraday's discoveries in Magnetic Electricity, and the other by Professor Turner on certain elementary points of Chemical Philosophy.

At ten o'clock on Thursday morning, the Vice-chancellor, attended by the heads of colleges and a numerous assemblage of doctors, entered the theatre, already crowded by members of the Association in the central area, ladies in the side, and under-graduates in the upper galleries; and, after some preliminary business, Dr. Phillimore, the professor of civil law, presented Sir D. Brewster, and Messrs. Brown, Faraday, and Dalton, to the Vice-chancellor, descanting in elegant Latin speeches on their respective merits, who then conferred upon them severally the degree of doctor of civil law; and they took their seats accordingly among the doctors, amidst the universal acclamations of the immense concourse assembled. Subsequently several members of the universities of Cambridge and Dublin were admitted *ad eundem.*

At twelve o'clock, about two hundred members, some on foot and some on horseback, met on and near Magdalen bridge, and formed, the equestrians a geological, the pedestrians a botanical expedition: at various points the two parties met, and several field lectures were given on geology by Dr. Buckland, and on botany by Professors Henslow and Burnett. After an excursion of about five hours, the parties returned, and the chief of the geologists dined with Dr. Buckland, the professor of geology, and of the botanists with Dr. Williams, the professor of botany.

In the evening the sections again met at the Clarendon, and various papers and reports were read, such as Dr. Knox on the Natural History of the Salmon, Sir T. Phillip on the Ascent of the Sap in Plants, &c., and which gave rise to some very animated discussion.

Friday morning the sections again assembled, and papers were read and lectures given by Mr. Vigors on the Geological Distribution of Animals, Dr. Danberry on the Geographical Distribution of Plants in the Neighbourhood of Oxford, (a previous paper having been read by Professor Henslow on the Distribution of Plants in Cambridgeshire,) and a paper communicated by Mr. Baxter, on the Spiral-like Fibres covering the Seeds of the Salvia Verbenaca and some other plants.

The remainder of Friday and Saturday were occupied in a similar manner to Tuesday and Wednesday, and the concluding lecture (on Geology) was, as we learn from a friend, delivered by Professor Buckland, who descanted with much eloquence on the Megatherium and other fossil remains; after which the session closed, with the announcement that the next meeting would be held at Cambridge. We much regretted our inability to remain until all was over: we left Oxford in the middle of the day on Friday, with feelings of much satisfaction at the proceedings and at the reception we had met with; and although we heard one or two objections made, we do not believe that any were well founded. We heard some complain that much of the matter reported on was not entirely novel; but they seemed to forget that the very object and nature of the reports was to give a summary of matters which had been discovered, and of the progress made by science during the past year, and that, although original communications were not excluded, still that such formed but a secondary object with the Association, which does not desire to occupy the ground already so well filled by numerous learned societies, but by its condensing their labours into a focus to assist and further the exertions of them all.

### METEOROLOGICAL JOURNAL,

By Messrs. Harris and Co. Mathematical Instrument Makers, 50, High Holborn.

| | Rain gauge. | Moon. | Thermometer. | | | Barometer. | | De Luc's Hygrometer. | | Winds. | | Atmospheric Variation. | | |
|---|---|---|---|---|---|---|---|---|---|---|---|---|---|---|
| June | | | 9a.m. | max. | min. | 9a.m. | 10p.m | 9a.m. | 10p.m. | 9 a.m. | 10p.m | 9 a. m. | 2 p.m. | 10 p.m. |
| 20 | | | 70 | 72 | 56 | 29.75 | 29.75 | 53 | 54 | NNW | NE | fine | fine | rain |
| 21 | .25 | ☽ | 63 | 72 | 57 | .71 | .63 | 54 | 56 | ENE | S | cloudy | | |
| 22 | | | 62 | 68 | 56 | .33 | .45 | 59 | 57 | S | W | rain | | fine |
| 23 | .45 | | 66 | 70 | 57 | .60 | .68 | 56 | 50 | WSW | WNW | fine | | |
| 24 | | | 64 | 67 | 55 | .74 | .81 | 50 | 50 | NW | NW | | | |
| 25 | | | 62 | 66 | 53 | .78 | .84 | 50 | 49 | NW | NW | showery | | |
| 26 | .04 | | 60 | 70 | 53 | .91 | .95 | 49 | 48 | NW | NNW | cloudy | | cloudy |
| 27 | | | 62 | 68 | 62 | 30.03 | 30.06 | 47 | 50 | NNW | NNE | fine | | fine |
| 28 | | ● | 68 | 73 | 62 | .13 | .12 | 51 | 50 | NE | SE | | | |
| 29 | | | 70 | 74 | 59 | .15 | .15 | 50 | 50 | NNE | SE | | | |
| 30 | | | 65 | 71 | 61 | .17 | .13 | 50 | 45 | E | SSE | | | |
| July 1 | | | 68 | 75 | 58 | .13 | .01 | 48 | 48 | SW | SW | | | |
| 2 | | | 70 | 75 | 57 | 29.97 | 29.95 | 46 | 47 | E | E | | | |
| 3 | | | 62 | 69 | 55 | .89 | .82 | 49 | 50 | E | SE | cloudy | | |
| 4 | | ☽ | 65 | 71 | 60 | .77 | .74 | 50 | 50 | SSE | NW | fine | | |
| 5 | | | 68 | 71 | 61 | .76 | .75 | 49 | 50 | NW | N | | | cloudy |
| 6 | .06 | | 69 | 73 | 57 | .67 | .55 | 50 | 51 | SW | SW | | | rain |
| 7 | | | 64 | 72 | 61 | .69 | .68 | 51 | 47 | NW | W | cloudy | | fine |
| 8 | | | 66 | 70 | 62 | .68 | .69 | 49 | 53 | SW | SW | fine | cloudy | cloudy |
| 9 | | | 68 | 73 | 62 | .70 | .77 | 53 | 52 | W | SW | | fine | |
| 10 | | | 68 | 69 | 62 | .72 | .65 | 52 | 54 | SW | SW | cloudy | | |
| 11 | | | 66 | 68 | 60 | .54 | .66 | 54 | 52 | SW | WNW | fine | | |
| 12 | | ○ | 68 | 76 | 57 | .65 | .50 | 52 | 54 | SW | NW | | | |
| 13 | | | 69 | 72 | 60 | .57 | .71 | 55 | 55 | WSW | SW | | | cloudy |
| 14 | | | 61 | 65 | 59 | .75 | 30.00 | 55 | 56 | SSE | N | rain | | fine |
| 15 | .94 | | 65 | 71 | 56 | 30.06 | .11 | 52 | 50 | NW | NW | fine | | |
| 16 | | | 67 | 72 | 62 | .02 | 29.93 | 50 | 52 | W | NW | cloudy | | |
| 17 | | | 70 | 78 | 62 | 29.93 | .81 | 52 | 50 | N | N | fine | | |
| 18 | | | 65 | 68 | 53 | .81 | .86 | 46 | 44 | NNW | NW | | | |
| 19 | | | 62 | 66 | 53 | .90 | .93 | 44 | 45 | NNE | NNE | | | overcast |

The quantity of Rain fallen in the month of June, was 2 inches and 74.100ths.

